# Supplementary material for: A framework for effective face-mask contact modeling based on finite element analysis for custom design of a facial mask
Source: PLoS One. 2022 Jul 21;17(7):e0270092. doi: 10.1371/journal.pone.0270092 (PMC9302758; doi:10.1371/journal.pone.0270092)
Supplement: Attachment — Submitted filename: 3. revision note.docx [file pone.0270092.s001.docx]

**Editor comment**

1. Please ensure that your manuscript meets PLOS ONE's style requirements, including those for file naming. The PLOS ONE style templates can be found at 
https://journals.plos.org/plosone/s/file?id=wjVg/PLOSOne_formatting_sample_main_body.pdf and 
https://journals.plos.org/plosone/s/file?id=ba62/PLOSOne_formatting_sample_title_authors_affiliations.pdf.

⇒ We have checked and corrected the style of the manuscript.

2. Please note that PLOS ONE has specific guidelines on code sharing for submissions in which author-generated code underpins the findings in the manuscript. In these cases, all author-generated code must be made available without restrictions upon publication of the work. Please review our guidelines at https://journals.plos.org/plosone/s/materials-and-software-sharing#loc-sharing-code and ensure that your code is shared in a way that follows best practice and facilitates reproducibility and reuse.

⇒ As we are preparing the technology for a commercial purpose, we would not want to share our system directly. But, mathematic algorithms for the core concept of our technology have been explained in the manuscript clearly and anyone in this field might easily understand and apply the concept in their studies.

3. Funding information should not appear in the Acknowledgments section or other areas of your manuscript. We will only publish funding information present in the Funding Statement section of the online submission form. Please remove any funding-related text from the manuscript and let us know how you would like to update your Funding Statement. Please include your amended statements within your cover letter; we will change the online submission form on your behalf.

⇒ The acknowledgement section is removed in the updated manuscript. And there’s no updates for the current Funding Statement.

4. We note that you have stated that you will provide repository information for your data at acceptance. Should your manuscript be accepted for publication, we will hold it until you provide the relevant accession numbers or DOIs necessary to access your data. If you wish to make changes to your Data Availability statement, please describe these changes in your cover letter and we will update your Data Availability statement to reflect the information you provide.

⇒ We have published our data resulting from the FE simulation shown in our study, and here is the DOI number: <http://dx.doi.org/10.13140/RG.2.2.21674.49607>.

**Reviewer 1**

1. The background and motivation need enhancement.

⇒ We have enhanced the background by making detailed descriptions in the introduction. More relevant studies have been added in the 3^rd^ paragraph of the introduction. The 4^th^ paragraph of the introduction has been newly added, and the 5^th^ paragraph of the introduction has been updated to emphasize our motivation for the study.

2. This paper's efficacy heavily depends on several assumptions. The simplified model is subsequently adopted, which reduces computational effort. I am curious about how the authors verify the result. I will suggest putting more emphasis on the verification part.

⇒ In our study, the verification was performed through an experiment of measuring the contact pressure using the FSR sensors, which is described in the Numerical Studies section. The values were compared using their mathematical order. Since the measured data was generated from the different masks and face shapes, we found that the contact pressure level and distribution of the algorithm are well-matched with the reference data. For example, the results of the contact pressure analysis in other papers indicate that the contact pressure shows a value between 0.001 MPa to 0.1 MPa in the cases of face-mask contact analysis found in the previous studies. And our results showed similar order to that obtained by the experiment in terms of the average (mean difference: 0.0005 MPa) and variation (range: 0.0087 to 0.0804 MPa), as discussed in the discussion section. However, as we tested for the one face through the pilot study, the similarity between the experimental and simulated results with various faces should be further studied to define the FE model more precisely. We newly added the 4th paragraph in the discussion to explain limitations and future studies.

3. Will the implanted electronics affect the contact performance?

⇒ Our proposed method simply calculates the contact pressure only between two surfaces (body surface and product surface) data without considering the mass of the human body or product. Therefore, the effect of the implanted electronics contacting inside of the human soft tissue is unavailable to estimate through our method.

4. Other scholars have also looked into mask-airway contact behaviours. For example, Liao H, et al. Sealing mechanism study of laryngeal mask airways via 3D modelling and finite element analysis. Scientific Reports. What are the difference, mainly the benefits, of using the approaches developed in this paper?

⇒ The goal of the presented method is to solve the face-mask contact problem efficiently using the simplified FE model and FE analysis procedure. Our method can compute the contact pressure of hundreds of cases in a short time. Thus, product designers can understand the contact pressure characteristics easily by changing design parameters. The quantified information resulting from our method can guide designers to obtain the best shape of a designated product quickly.

5. The paper needs thorough proofreading to remove the grammatical error.

⇒ We found the grammatical errors and the manuscript was proofread.

**Reviewer 2 comment**

1. The authors performed “FE model of the face” but I couldn’t see FE model outputs (stress and deformation) in manuscript. They should be showed the outputs.

⇒ The displacement and stress commonly used as estimators in FE analysis were considered in our mathematic process (equation 1); but, we used the contact pressure as an indicator of discomfort, air leakage, and usability. As the FE model can be verified by using the contact pressure as it can also be measured by sensors in a real situation. Also, this study didn’t calculate the deformation to reduce the computational load in the proposed procedure. Therefore, displacement and stress were not used in our framework. The following figure shows stress, displacement, and contact pressure showing a similar trend. We have added about this issue in the 5^th^ paragraph of the introduction and the 1^st^ paragraph of the discussion sections.

2. Today, computer aided finite element analysis (FEA) was used to solve processes simulations of COVID-19 and other infections and biomechanics [1-5]. The authors should be cited the following references to enrich of paper.

⇒ We have cited the mentioned papers in the introduction.
